# Epidemiology of enterotoxigenic *Escherichia coli* among children under five years in Kenya’s urban informal settlement

**DOI:** 10.3389/fmicb.2025.1637369

**Published:** 2025-08-15

**Authors:** Amos Njuguna, John M. Maingi, Cecilia Mbae, Phelgona Otieno, Kelvin Kering, Winfred Mbithi, Christine Kioko, Rahma Osman, Evans Kibet, Samuel Kariuki

**Affiliations:** ^1^Department of Microbiology, Biochemistry and Biotechnology, Kenyatta University, Nairobi, Kenya; ^2^Center for Microbiology Research, Kenya Medical Research Institute, Nairobi, Kenya; ^3^Drugs for Neglected Diseases Initiative, Nairobi, Kenya

**Keywords:** ETEC, diarrhea, children, informal settlement, Kenya

## Abstract

**Introduction:**

Enterotoxigenic *Escherichia coli* (ETEC) is a significant cause of diarrheal morbidity and mortality among children under 5 years, particularly in low and middle-income countries. This study aimed to determine the prevalence of ETEC and associated risk factors among children under five presenting with diarrhea in health facilities in Mukuru slums of Nairobi, Kenya, where poor sanitation and hygiene practices are prevalent.

**Methods:**

Using a cross-sectional design, we recruited 387 children under five years of age with acute diarrhea. Total nucleic acid (TNA) was extracted from stool samples and analyzed using a PCR-based customized TaqMan Array Card (TAC), which included three ETEC toxin genes (LT, STh, and STp) and six ETEC colonization factors (CFs). A structured questionnaire was employed to collect participants information.

**Findings:**

ETEC was detected in 148/387 samples, yielding a prevalence of 38.2% (95% CI: 34.2–42.2%). Both heat-labile and heat-stable (LT/ST) enterotoxin genes were the most common (43%) followed by heat-labile (30%), and heat-stable (27%). Colonization factors (CF) were present in 52% (77/148) of ETEC-positive samples with CS6 being the most frequently detected CF at 36.3% (28/77). Children aged 13–24 months had significantly elevated odds of infection (aOR = 2.48; 95% CI: 1.36–4.52, *p* = 0.003), as well as those aged 25–36 month (aOR = 2.12; 95% CI: 1.10–4.10, *p* = 0.025), 37–48 months (aOR = 2.45; 95% CI: 1.21–4.97, *p* = 0.013), and 49–59 months (aOR = 2.59; 95% CI: 1.12–6.01, *p* = 0.027). Households lacking access to private flush toilets exhibited a tripled risk (aOR = 3.04; 95% CI: 1.38–6.71, *p* = 0.006) of ETEC positivity.

**Conclusion:**

These findings highlight the urgent need for targeted public health interventions, including improved sanitation and hygiene practices and vaccine introduction, to mitigate the burden of ETEC-related diarrhea among high-risk populations in disease endemic settings.

## 1 Introduction

ETEC is often the first bacterial pathogen responsible for diarrhea in infants and children and is responsible for two to five diarrheal episodes during the first 3 years of life (Kotloff et al., 2013). Globally, it is estimated that ETEC causes 220 million cases and over 50,000 deaths annually, with a huge proportion of the cases and mortalities affecting children under 5 years in developing countries (Khalil et al., 2018). ETEC is typically transmitted through contaminated food and water exposed to human waste (Gonzales-Siles and Sjöling, 2016), with direct person-to-person spread rare due to its high infectious dose. Poor hygiene practices during primary caregiving can become a significant source of contamination for the food consumed by children (Roussel et al., 2017). In countries where ETEC is endemic, children are often infected multiple times early in life. In addition, ETEC infection has been associated with most traveler’s diarrhea among visitors to endemic developing countries, causing an estimated 10 million cases of diarrhea annually (Bourgeois et al., 2016).

ETEC express one or both of two types of enterotoxins; heat-stable (ST) and/or heat-labile (LT) toxins which are encoded by the same or separate plasmids and may produce one or more of several colonization factors (CFs) that promote adherence to and colonization of the host intestinal epithelium (Ifeanyi et al., 2018). Epidemiological studies of childhood diarrhea have found an association between infections with CF-positive ETEC and diarrhea, but for the individual CFs, this association seems uncertain. In addition, previous studies undertaken in different geographical areas have reported substantial variation in the distribution of toxins and CFs on ETEC isolates (Mcconnell et al., 1991; Sommerfelt et al., 1996). As a result, there is growing interest in the development of vaccines as a more expedient solution to prevent ETEC infection. To provide broad-spectrum protection, an ETEC vaccine should contain fimbrial antigens representative of prevalent ETEC pathogens (Qadri et al., 2005). Since CFA/I and CS1 to CS6 are the most common human ETEC fimbriae, they are key immunogen candidates in an ETEC vaccine (Gaastra and Svennerholm, 1996).

Although several studies (Kipkirui et al., 2021; Iijima et al., 2017; Makobe et al., 2012; Mbuthia et al., 2018; Dimbuson Bulimo et al., 2014) have reported on the burden of ETEC in children under 5 years old there is need for more data on the most common CFs and enterotoxins present in ETEC. Therefore, this study aimed to determine the prevalence of ETEC and associated risk factors among children under five years in a low-resource urban informal settlement in Nairobi, Kenya. This information is critical in the development of intervention strategies, including designing vaccine targets and providing baseline information for further research.

## 2 Materials and methods

### 2.1 Study site and population

The study was conducted in Mukuru, one of the urban informal settlements in Nairobi, located about 15 kilometers east of the city. This informal settlement, which is home to approximately 700,000 residents (Kenya National Bureau of Statistics Ministry of State for Planning, National Development and Vision 2030, 2012) face significant public health challenges, which are associated with inadequate access to clean water, overcrowding, poor sanitation, and open sewers, that facilitate the rapid spread of infectious diseases (Mbae et al., 2020). Although Mukuru is subdivided into eight villages, this study focused on the two most populous villages: Mukuru Kwa Reuben and Mukuru Kwa Njenga.

### 2.2 Study participants, sample, and data collection

Eligible participants were children presenting with acute diarrhea at four primary healthcare facilities; Medical Missionaries of Mary Clinic, Mukuru City Council Clinic, Njenga Level IV Hospital, and Reuben Health Centre. Participants were enrolled into the study after written informed consent was obtained from parents/guardians.

Stool samples or rectal swabs were collected and transported to microbiology labs at the Kenya Medical Research Institute where they were stored at −80°C until DNA extraction.

A structured questionnaire was used to collect information on socioeconomic; demographic and household WASH characteristics of the children recruited for the study.

### 2.3 Laboratory procedures

#### 2.3.1 TNA extraction

The QIAamp Fast DNA Stool Mini Kit (QIAGEN, Hilden, Germany) was used to extract total nucleic acid (TNA) from whole stool samples and swabs (QIAGEN, 2020). Two extrinsic controls, Phocine Herpesvirus (PhHV) and bacteriophage MS2 (QIAGEN, Valencia, CA, United States), were used to monitor extraction and amplification efficiency. An extraction blank containing extrinsic controls, nuclease-free water and lysis buffer with no sample was incorporated during all extractions control for laboratory contamination (Liu et al., 2016). To achieve a homogeneous mixture, approximately 370 mg of glass beads (Sigma-Aldrich, St. Louis, MO, and Burlington, MA, United States) were added to 180–220 mg of stool or swab before introducing the lysis buffer. The extraction process was done as per the manufacturer’s instructions (QIAGEN, 2020). The TNA was aliquoted into two 1.5 mL microcentrifuge tubes each contains 100 μL, which were then stored at −80°C.

#### 2.3.2 Detection of ETEC enterotoxin genes and colonization factors genes

Detection of ETEC toxins and colonization factors was undertaken through a TaqMan Array Card platform (Thermo Fisher, Carlsbad, CA, United States) (Liu et al., 2016). Quality control was performed in accordance with the manufacturer’s guidelines on TAC analysis (Applied Biosystems, 2009). The TNA was tested for the ETEC enterotoxin genes (*eltB1, estA1, and estA2–4*) (Kipkirui et al., 2021) and six colonization factors, including CFA/I and CS1, CS2, CS3, CS5 and CS6 considered as most commonly found human ETEC fimbriae (Gaastra and Svennerholm, 1996). The platform amplified the DNA for the ETEC toxins and colonization factors, and the amplification curves were analyzed using the QuantiStudio7 veriflex software (version 1.2.4) (Liu et al., 2016). The 100 μL PCR reaction composed of 50 μL of Ag-Path-ID 2X RT-PCR buffer, 4 μL of Ag-Path-ID Enzyme mix, and 20 μL of total nucleic acid extract from stool specimens supplemented with 26 μL of nuclease-free water or 46 μL of total nucleic acid extract from rectal swabs. All reagents were from Thermo Fisher, Carlsbad, CA, USA. The real-time PCR was performed with the cycling condition of 20 min at 45°C, 10 min at 95°C, 40 cycles of 15 s at 95°C, and 1 min at 60°C (Liu et al., 2025). ETEC was defined as the detection of either LT, STh, or STp genes. Any sample with cycle threshold (Ct) < 35 was considered positive for the ETEC toxin and colonization factors. A blank control and the detection of extrinsic controls were utilized to validate sample results in PCR reactions and to accurately evaluate the pathogen target PCR results, respectively. The extraction blank was considered valid only if it tested positive for PhHV and negative for any ETEC toxin and colonization factor. If any ETEC toxin and colonization factor was detected in an extraction blank, it indicated a contamination issue. Consequently, positive results for that target in any samples extracted within that batch were deemed invalid. The detection of PhHV in the sample confirmed the successful extraction and amplification of DNA. Conversely, if PhHV was not detected, the results for any undetected ETEC toxin and colonization factor in that sample were rejected, rendering those negative DNA target results invalid.

### 2.4 Data analysis and management

The laboratory analysis results were entered into the Epicollect 5 software and subsequently exported to a password-protected Excel sheet, accessible only to authorized personnel. Statistical analyses were conducted using STATA version 15.1. The data underwent cleaning in STATA by running frequency analyses for various variables. Consistency checks were performed to identify and rectify any inconsistencies in the data, including duplicate entries, which were removed when found. Descriptive statistics and frequency tables were employed to summarize the data. For the socio-demographic description of participants, proportions and frequencies were calculated, while continuous variables were summarized using the mean. The prevalence of ETEC was determined as the proportion of children who tested positive for ETEC.

Sociodemographic characteristics, health behavioral and environmental factors were regarded as independent variables, with ETEC positivity as the dependent variable. A Chi-square test was conducted to analyze differences in the proportion of ETEC-positive samples among various age groups and to examine associations between colonization factors and toxin type. Bivariate and multivariable analyses, were conducted to examine the associations between the dependent and independent variables. A univariable binary logistic regression analysis was performed to identify potential predictors for inclusion in the multivariable binary logistic regression model. The crude odds ratio (cOR) for each independent variable was calculated. To evaluate multicollinearity among the independent variables, the variance inflation factor (VIF) was calculated, resulting in the exclusion of predictors with VIF values exceeding 5 from the initial model. Subsequently, variables with a *p*-value less than 0.20 from the univariable analysis were analyzed in a multivariable framework to control for potential confounding factors. The multivariable model was constructed using a backward stepwise selection algorithm, which iteratively removed predictors until no further improvements in model fit were observed. The goodness of fit for the final model was evaluated using the Hosmer-Lemeshow test, which indicated a satisfactory fit (*p* = 0.5693). The strength of the associations was expressed as adjusted odds ratios (AOR) with 95% confidence intervals (CI), with a *p*-value of < 0.05 considered statistically significant. The results are presented through clearly structured tables, figures, and narrative descriptions to enhance clarity and understanding.

### 2.5 Ethical consideration

Ethical approval to undertake this study was obtained from KEMRI’s Scientific Ethics and Review Unit (SERU) (KEMRI/SERU/CMR/P00276-04-2024/4990). In addition, a study permit was obtained from the National Commission for Science, Technology, and Innovation (NACOSTI) (NACOSTI/P/24/38888).

## 3 Results

### 3.1 Frequency of ETEC diarrhea

The age distribution of participants ranged between 1 month to 59 months. The median age was 25 months while the mode was 9 months. Majority (28.4%, 110/387) of the children presenting with diarrhoea were aged 13–24 months (Table 1). More than half (55.8%, 216/387) of the children presenting with diarrhea were ≤ 2 years. In addition, a huge proportion (53.7%, 208/387) of the patients were males. Of the 387 stool samples, ETEC was detected in 148 (38.2, 95% CI: 33.4–43.3) samples. The prevalence of ETEC infection was slightly higher in female subjects (39.7%; 71/179), compared to male (37.0%; 77/208) though this variation was not statistically significant (*p* = 0.593). The fraction of ETEC detected were almost similar in each age group beside in children aged ≤12 months where ETEC detection were low and with a majority detected in children aged 13–24 months. There was significant difference in the distribution of ETEC across age groups (*p* = 0.017) as shown in Table 1. Out of the 148 ETEC positive samples, both heat labile and heat stable (LT/ST) enterotoxin were detected in 43% (64/148) of the samples, followed by heat-labile (LT) alone (30%, 44/148), and heat-stable (ST) toxin (27%,40/148). The frequency of STh, STp and STh + STp in ST-ETEC was 53.9% (56/104), 34.6% (36/104) STp, and 11.5% (12/104) respectively (Table 2).

**Table 1:** Comparison of age and gender of participants with ETEC test results using Chi-square test.

Diarrheic subjects	Number (%)	ETEC-positive patients no. (%)	*P*-value
Age (months)			0.017
≤12	106	26 (24.5)	
13–24	110	50 (45.5)	
25–36	75	32 (43.7)	
37–48	62	25 (40.3)	
49–59	34	15 (44.1)	
Gender			0.593
Female	179	71 (39.7)	
Male	208	77 (37.0)	

**Table 2:** Distribution of ETEC toxins in positive samples.

Toxin	LT	ST	LT/ST	STh/STp		
				STh	STp	STh/STp
Positives	44/148	40/148	64/148	56/104	36/104	12/104
Percentage %	29.7%	27%	43.2%	53.9%	34.6%	11.5%

### 3.2 Detection of CFs

CF genes were detected in 52% (77/148) of ETEC positive samples. CS6 was the most common CF (36.3%,28/77), followed by CFA/1 (23.4%, 18/77), a combination of CS2 & CS3 genes at 9.1% (7/77), CS1 5.2% (4/77), combination of CS2 & CS3 and CFA/1 & CS6 at 3.9%(3/77), CS5, CS3, and combination of CS1 & CS3, and CFA/1 & CS1 & CS2 & CS3 & CS6 at 2.6% (2/77) and CS2, combination of CS1 & CS3, CS1 & CS6, CFA/1 & CS2 & CS3 & CS6, CFA/1 & CS1, and CFA/1 & CS1 & CS3 & CS6 all equally at 1.3% (1/77) (Table 3).

**Table 3:** Distribution of colonization factors in ETEC positive samples.

No.	CFA/1	CS1	CS2	CS3	CS5	CS6	Total detection in ETEC positives samples
1	Negative	Negative	Negative	Negative	Negative	Positive	28
2	Negative	Negative	Negative	Negative	Positive	Positive	2
3	Negative	Negative	Negative	Positive	Negative	Negative	2
4	Negative	Negative	Positive	Negative	Negative	Negative	1
5	Negative	Negative	Positive	Positive	Negative	Negative	7
6	Negative	Negative	Positive	Positive	Negative	Positive	3
7	Negative	Positive	Negative	Negative	Negative	Negative	4
8	Negative	Positive	Negative	Positive	Negative	Negative	1
9	Negative	Positive	Negative	Negative	Negative	Positive	1
10	Negative	Positive	Negative	Positive	Negative	Positive	2
11	Positive	Negative	Negative	Negative	Negative	Negative	18
12	Positive	Negative	Negative	Negative	Negative	Positive	3
13	Positive	Negative	Positive	Positive	Negative	Positive	1
14	Positive	Positive	Negative	Negative	Negative	Negative	1
15	Positive	Positive	Negative	Positive	Negative	Positive	1
16	Positive	Positive	Positive	Positive	Negative	Positive	2
Total							77

### 3.3 Association of CFs with toxin types

**Figure 1:**
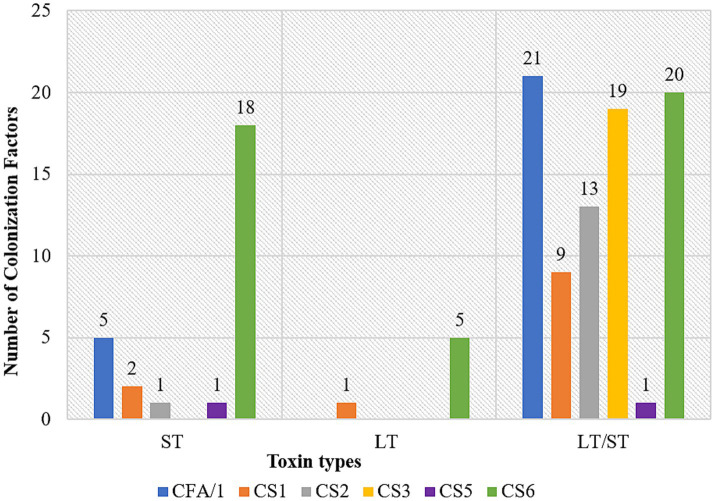
Association of CFs with toxin types; ETEC heat stable (ST), ETEC heat labile (LT) and ETEC heat labile/heat stable (LT/ST) along X-axis and number of colonization factors (CF) on the Y-axis.

CFs genes association differed by ETEC enterotoxin. In LT/ST, the CF genes were detected in 59.7% (46/77), indicating a significant association (*p < 0.001*). Similarly, the ST toxin type also demonstrated a significant association, with 32.5% (25 out of 77) CF genes detected (*p < 0.001*). LT toxin type showed no significant association with 7.8% (6/77) CF genes detected (*p = 0.269*) (Table 4). In ST, CS6 was majorly detected. CS3 gene was not detected in ST. In LT, CS6 and CS1 were the only detected CF genes. In LT/ST, all the tested CF genes were detected. CFA/1 gene was majorly detected while CS5 was the least detected (Figure 1). Among the 71/148 (48%) positive identified ETEC that lacked an identifiable CF, 38/71 (53.5%) were LT, 18/71 (25.4%) were LT/ST, and 15/71 (21.1%) were ST.

**Table 4:** Association of CFs with toxin types analyzed by Chi-square test.

Toxin type	Positive with zero CF	Positives with CF	*P*-value
LT/ST	18 (25.4%)	46 (59.7%)	0.000
ST	15 (21.1%)	25 (32.5%)	0.000
LT	38 (53.5%)	6 (7.8%)	0.269

### 3.4 Risk factors analysis

#### 3.4.1 Sociodemographic characteristics of household

Among the households surveyed, 48% had 2 to 3 family members, with a proportion of 37.1% of children testing positive for ETEC. Additionally, 45% of households had 4 to 5 members, with a proportion of 41.4% of children testing positive for ETEC. Notably, 83% of households had at least one child under five, with a proportion of 38% of these children testing positive for ETEC. In terms of education, 22.2% of household heads had primary education or less, with a proportion of 44.2% of children from these households testing positive for ETEC. The majority of households (56.3%) used corrugated iron for their main structure, with a proportion of 43.1% of children in these households testing positive. Cooking fuel varied, with gas being the most common at 72.4%; however, households using charcoal had the highest proportion of children testing positive for ETEC at 56.7%. Monthly expenditures indicated that 31% of households spent Ksh 9,900 or less, with a proportion of 44.2% of children testing positive in these households. Ownership of electricity was high at 79.6%, but only 3.9% owned a refrigerator, with low proportions of ETEC positivity in children from these households. Additionally, the duration of residence affected positivity, with a proportion of 34.5% of children in households living for less than two years testing positive for ETEC (Table 5).

**Table 5:** Household characteristics.

Sociodemographic characteristics of household	*N* = 387	%	ETEC positives	
			*N* = 148%	
Number of family members in the household				
2 to 3	186	48	69	37.1
4 to 5	174	45	72	41.4
6 and above	27	7	7	25.9
Number of children less than 5 years old				
1	321	83	122	38
2	60	15.5	25	41.7
3 to 4	6	1.5	1	16.7
Number of children above 5 years old				
0	212	54.8	78	36.8
1	118	30.5	51	43.2
2 to 4	57	14.7	19	33.3
Education of the household head				
Primary school and below	86	22.2	38	44.2
Junior high school/technical secondary	230	54.4	88	38.3
College and above	71	18.4	22	31
Main composition of households				
Corrugated Iron	218	56.3	94	43.1
Masonry	169	43.7	54	32
Rooms are used by household				
1	341	88.1	132	38.7
2 and above	46	11.9	16	34.8
Main fuel used for cooking by the household				
Charcoal	30	7.7	17	56.7
Gas	280	72.4	98	35
Kerosene	77	19.9	33	42.9
Average expenditure per month of household				
Ksh 9,900 and below	120	31	53	44.2
Ksh 10,000–Ksh. 24,900	184	47.6	73	39.7
Ksh 25,000 and above	83	21.4	22	26.5
Households’ ownership of selected items				
Electricity	308	79.6	116	37.7
Sofa	278	71.8	106	38.1
Telephone	219	56.6	75	34.2
Sewing machine	10	2.6	5	50
Refrigerator	15	3.9	3	20
Duration household members lived in the area				
Less than 2 Years	133	34.5	46	34.6
2–5 years	190	49.1	74	38.9
5 years and above	64	16.4	28	43.8

#### 3.4.2 Household distribution by water hygiene, sanitation, source of food and domestic animal kept

The proportion of children with ETEC was 39.0% in households that relied on communal or municipal water (82.2%); whereas the proportion of children from households using untreated water sources, such as boreholes (1.3%), had the highest positivity rate at 60%, though this was based on just 5 cases. Water treatment was practiced by 41.9% of households, with 45.3% of children (*n* = 24/53) among those using Aqua tabs testing positive for ETEC. Among households that stored water in jerry cans (93.8% usage), 39.1% of children had ETEC, while those with direct tap access (6.98%) showed a lower prevalence of 22.2%. Sanitation infrastructure also played a critical role; 45.0% of children in households using shared pit latrines (25.8%) had ETEC, compared to only 18.0% among those with private flush toilets (12.9%). Hygiene behaviors revealed paradoxes: households reporting “sometimes” washing hands after defecation (35.6%) had a higher proportion of children with ETEC at 42.8% compared to consistent washers (35.5%). Additionally, frequent street food consumption (≥6 times/week, 39.5% of households) showed a dose–response relationship, with a proportion of children with ETEC at 43.8% compared to 30.9% for those consuming street food 1–2 times/week. The proportion of children with ETEC was 38.8% among households near contamination sources (81.9%). Animal husbandry further modulated risk, with the proportion of children with ETEC at 75.0% in households that kept cattle (1.0%) and 40.4% in those that kept cats (48.6%) (Table 6).

**Table 6:** Household distribution by water hygiene, sanitation, source of food and domestic animal kept.

Variables	*N* = 387	%	ETEC positives	
			*N* = 148	%
Main source of drinking water in the house				
Borehole	5	1.29	3	60
Bottled water	52	13.44	18	34.6
Communal/ Municipal tap	318	82.17	124	39
Water Vendor	12	3.1	3	25
Households that treat water	162	41.9	65	40.1
Methods used to treat water before drinking				
Boiling	77	19.9	29	37.7
Aqua tabs	53	13.7	24	45.3
Filtration	33	8.53	13	39.4
Types of water storage used in the home				
Directly from tap	27	6.98	6	22.2
Bucket	44	11.37	12	27.3
Drum	125	32.3	38	30.4
Jerri can	363	93.8	142	39.1
Type of toilet used by the household				
Shared flush toilet	193	49.9	78	40.4
Private Flush toilet	50	12.9	9	18
Private Pit latrine	2	0.52	2	100
Bush/river/canal	1	0.26	0	0
Shared pit latrine	100	25.8	45	45
carrier bags	1	0.26	0	0
Public toilet (Fresh life)	45	11.6	16	35.6
Household members wash hands after defecation				
Always	248	64.1	88	35.5
Sometimes	138	35.6	59	42.8
Never	1	0.3	1	100
Household members wash hands before food preparation				
Always	217	56.1	89	41
Sometimes	154	39.8	53	34.4
Never	16	4.1	6	37.5
Household members wash hands before eating				
Always	312	80.6	121	38.8
Sometimes	75	19.4	27	36
Frequency of family eating street food				
1 to 2 times/week	97	25.1	30	30.9
3–5 times/week	120	31	47	39.2
6 or more/week	153	39.5	67	43.8
Never	17	4.4	4	23.5
Household members eat kachumbari	90	23.3	34	37.8
Source of household’s vegetables				
Buy from shop	2	0.5	0	0
Buy from neighbor	1	0.3	0	0
Buy from our mobile vendor/mama mboga	356	92	133	37.4
Buy from village market	59	15.3	23	39
Existing contamination sources around the water source within 20 m	317	81.91	123	38.8
e.g. open sewers, communal latrines/toilets				
Domestic animals kept by households’ members				
Cattle	4	1	3	75
Dogs	39	10.1	17	43.6
Pigs	18	4.7	9	50
Cats	188	48.6	76	40.4
Chicken	74	19.2	33	44.6
Goats	17	4.4	8	47.1
Turkey	3	0.8	1	33.3
Dove	2	0.5	0	0
Duck	17	4.4	5	29.4
Sheep	3	0.8	2	66.7

#### 3.4.3 Factors associated with ETEC infection (bivariate analysis)

This study identifies several demographics, behavioral, socioeconomic, and environmental predictors of ETEC infection in a resource-limited urban population. Age was strongly associated with ETEC, with children aged 13–24 months exhibiting the highest odds (OR = 2.56, 95% CI = 1.44–4.58, *p* = 0.001), followed by sustained elevated risks through 49–59 months (OR = 2.43, 95% CI = 1.08–5.45, *p* = 0.031). Structural determinants played a critical role: households with masonry walls (OR = 0.61, 95% CI = 0.41–0.94, *p* = 0.025), private flush toilets (OR = 0.31, 95% CI = 0.15–0.66, *p* = 0.002) and drum-based water storage (OR = 0.60, 95% CI = 0.38–0.95, *p* = 0.029) demonstrated significant protection. Behavioral exposures revealed that households using gas for cooking show a significant infection risk reduction (OR = 0.41, 95% CI = 0.192–0.883, *p* = 0.023) compared to charcoal. Non-significant trends suggested potential risks with shared pit latrines (OR = 1.46, 95% CI = 0.92–2.32, *p* = 0.107), and lower household head education (OR = 1.76 for primary schooling, 95% CI = 0.91–3.41, *p* = 0.092). Protective trends emerged for refrigerator access (OR = 0.39, 95% CI = 0.11–1.41, *p* = 0.152) though this lacked statistical significance. Direct tap water use demonstrated marginal protection (OR = 0.44, 95% CI = 0.17–1.11, *p* = 0.083) (Supplementary Table 1).

#### 3.4.4 Multivariate analysis

After adjustment for potential confounders, key predictors of ETEC infection were determined. Compared to infants aged ≤12 months (ref), significantly elevated odds were observed in children aged 13–24 months (aOR = 2.48; 95% CI: 1.36–4.52, *p* = 0.003), 25–36 month (aOR = 2.12; 95% CI: 1.10–4.10, *p* = 0.025), 37–48 months (aOR = 2.45; 95% CI: 1.21–4.97, *p* = 0.013), and 49–59 months (aOR = 2.59; 95% CI: 1.12–6.01, *p* = 0.027). Households without access to private flush toilets exhibited a tripled risk compared to those with such facilities (aOR = 3.04; 95% CI: 1.38–6.71, *p* = 0.006). Households using charcoal as primary cooking fuel had 2.52-fold higher infection odds compared to gas users (95% CI: 1.13–5.61, *p* = 0.024), whereas kerosene use showed no association (aOR = 1.14; 95% CI: 0.66–1.95, *p* = 0.636) (Table 7).

**Table 7:** Results from multivariate logistic regression model (AOR) (*p* < 0.2).

Factor	Adjusted odds ratio	*P*-value	95% conf. interval
Age, months			
≤12	Ref		
13–24	2.47903	0.003	1.358333–4.524349
25–36	2.12447	0.025	1.100016–4.102985
37–48	2.45398	0.013	1.211336–4.971384
49–59	2.59001	0.027	1.116564–6.007834
Main fuel			
Gas	Ref		
Charcoal	2.51985	0.024	1.132404–5.607218
Kerosene	1.13911	0.636	0.6639312–1.954367
Use private flush toilet			
Yes	Ref		
No	3.03882	0.006	1.37684–6.706964
Use bucket			
No	Ref		
Yes	0.56384	0.128	0.2698557–1.178103
Wash hands before food prep			
Never	Ref		
Always	1.35678	0.581	0.4584961–4.014981
Sometimes	0.86229	0.792	0.2868898–2.591724

## 4 Discussion

ETEC is a major etiological agent of diarrhea in children under five in developing countries. It is often the first or second most commonly isolated pathogen in Africa, Asia, Latin America, and the Caribbean (Svennerholm, 2011). In our study, the prevalence of ETEC among children under five years of age with acute diarrhea was found to be 38.2%. This finding is notably higher than the proportions previously reported in Kenya, which ranged from 4.3 to 29.8% (Kipkirui et al., 2021; Iijima et al., 2017; Makobe et al., 2012; Mbuthia et al., 2018; Dimbuson Bulimo et al., 2014). Findings from various studies conducted in different regions of the world have reported lower prevalence rates compared to the present findings (Ifeanyi et al., 2018; Thakur et al., 2018; Subekti et al., 2003; Rasul et al., 2022). The discrepancies between our findings and those from previous studies may arise from variations in testing methodologies. Our study utilized TAC, whereas the other contrasting studies relied on conventional culture-based techniques. The use of molecular diagnostic methods, such as TAC, has been shown to significantly enhance the detection of ETEC compared to conventional culture approaches (Liu et al., 2025). Also, the discrepancies may be attributed to geographical factors, and differences in target populations.

The LT/ST toxin genes were the most frequently detected at 43%. This finding is consistent with findings from studies conducted in Bangladesh and India, which reported LT/ST as the most common toxin genes (Begum et al., 2016; Rivera et al., 2023; Nunes et al., 2011). Discrepancies in the predominant toxin types have been observed in various studies conducted in Kenya. One study identified LT toxin as the most prevalent enterotoxin (Iijima et al., 2017), while another reported ST toxin as the predominant type (Kipkirui et al., 2021). However, these findings contrast with studies from Egypt and Peru, where LT was identified as the most common toxin (Rivera et al., 2023; Mansour et al., 2014). Additionally, a study from Indonesia indicated that ST was the most prevalent toxin (Subekti et al., 2003).

CF genes were detected in 52% of ETEC-positive strains, which is comparable to findings from studies in Nicaragua and Brazil, where detection rates were 50% (Nunes et al., 2011; Vilchez et al., 2014). However, this prevalence is lower than that reported in a study in Kenya, which documented a rate of 60.9% (Kipkirui et al., 2021). In contrast, our findings exceed those from another study in Kenya, which reported a CF gene detection rate of 46.9% (Dimbuson Bulimo et al., 2014). Furthermore, our results are higher than those observed in studies conducted in Egypt (33%) and Nigeria (43.7%) (Ifeanyi et al., 2018; Mansour et al., 2014). In our study, CS6 emerged as the most frequently detected CF. This finding is consistent with reports from various studies in LMICs that identify CS6 as the predominant CF (Kipkirui et al., 2021; Liu et al., 2025; Simuyandi et al., 2019; Qadri et al., 2000). This contrasts with a study in Kenya, where CFA/I was the most prevalent CF (Dimbuson Bulimo et al., 2014), while CS2 and CFA/I were found to be the most common CFs in Nigeria and Egypt, respectively (Ifeanyi et al., 2018; Mansour et al., 2014). The discrepancies between our findings and those from other studies may be attributed to variations in testing methodologies used for CF identification, as well as differences in CF expression by ETEC across geographical regions (Nunes et al., 2011). Furthermore, the loss of plasmids harboring CFs could potentially contribute to these observed differences (Dimbuson Bulimo et al., 2014).

The present findings indicate that LT/ST followed by ST are most likely to express a colonization factor, aligning with reports from diverse geographical regions (Qadri et al., 2000; Wolf, 1997). While the formulation of ETEC vaccines predominantly relies on LT toxins and prevalent CFs (McKenzie et al., 2007), the current findings, along with existing literature linking ST-positive ETEC to symptomatic disease (Qadri et al., 2000; Clemens et al., 2004), underscore the necessity of integrating a comprehensive approach in the development of effective ETEC vaccine strategies. It is imperative to combine highly antigenic vaccine formulations with optimized delivery regimens. Sole reliance on LT-mediated antitoxic immunity may prove insufficient, particularly in regions where ST-only ETEC strains are endemic. This comprehensive strategy aims to elicit a robust immune response against ETEC strains that disproportionately affect infants and children in developing countries (Walker et al., 2007).

In this study, 48% of detected ETEC exhibited no identifiable CFs. These results align with findings from studies conducted in Kenya (Kipkirui et al., 2021; Dimbuson Bulimo et al., 2014) and corroborate previous research indicating that 30 to 50% of ETEC strains globally do not express detectable CFs (Qadri et al., 2000; Rodas et al., 2011; Rivera et al., 2010). The absence of CFs has primarily been associated with LT strains (Ifeanyi et al., 2018; Dimbuson Bulimo et al., 2014; Shaheen et al., 2004) which is consistent with our results where 51.9% of the LT strains lacked a detectable CF. However, some studies have reported that CFs is nearly equally associated with LT and ST-positive ETEC strains (Ifeanyi et al., 2018; Nunes et al., 2011). The inability to identify a CF may be attributed to the expression of CFs not targeted by the primers used in the TAC-PCR panel, as at least 29 distinct CFs have been identified (von Mentzer and Svennerholm, 2024), as well as the presence of unidentified CFs that remain uncharacterized (Ifeanyi et al., 2018).

The observed significantly elevated odds of ETEC infection in children aged 13–24 months (aOR = 2.48, *p* = 0.003), 25–36 months (aOR = 2.12, *p* = 0.025), 37–48 months (aOR = 2.45, *p* = 0.013), and 49–59 months (aOR = 2.59, *p* = 0.027) compared to infants aged ≤12 months reflect age-dependent exposure patterns prevalent in low-resource settings corroborating findings from other regions (Wilunda, 2006; Boadi and Kuitunen, 2005; Dewey and Adu-Afarwuah, 2008). This increased susceptibility can be attributed to several factors, including heightened mobility and inquisitiveness. As children become more active, they explore their environment more extensively, often placing objects in their mouths, which can lead to increased exposure to pathogens and contaminants (Gorman Ng et al., 2016; Xue et al., 2007). Additionally, this developmental stage is marked by the introduction of complementary feeding, which can expose children to contaminated food and water sources. The transition from a milk-based diet to solid foods, coupled with declining maternal antibodies, further compounds the risk of gastrointestinal infections in this age group (Checkley et al., 2008).

Households using charcoal as their primary cooking fuel exhibited 2.52 times greater odds of ETEC infection compared to those utilizing gas (*p* = 0.024). The reliance on charcoal as a solid fuel is often an indicator for poor socioeconomic status among households, which may lack access to cleaner energy alternatives (Rasel et al., 2024).

Households lacking access to private flush toilets exhibited an increased risk of ETEC, with an adjusted odds ratio (aOR) of 3.04 (*p* = 0.006). This heightened risk is largely attributable to the use of shared flush toilets and latrines, which can facilitate frequent contact with contaminated surfaces harboring ETEC pathogens from multiple users. Inadequate cleaning and maintenance of these communal facilities may lead to the accumulation of ETEC pathogens, thereby increasing the likelihood of disease transmission. The presence of private flush toilets offers a protective benefit, as enhanced sanitation directly blocks pathways of exposure, significantly reducing infection risk (Fewtrell et al., 2005). These findings align with previous research demonstrating that improved sanitation is associated with reduced rates of diarrheal diseases (Fewtrell et al., 2005; Montgomery and Elimelech, 2007).

Limitations of this study include the examination of only six ETEC colonization factors, with additional colonization factors not included in the TAC. Additionally, utilizing a cross-sectional design allowed us to identify associations between various factors and ETEC infection; however, this approach limits causal inference. Future research should adopt longitudinal designs to better investigate causality and how variables evolve over time, thereby enhancing our understanding of ETEC infection dynamics. These findings highlight the urgent need for targeted public health interventions, including improved water supply through access to clean water and regular water quality testing, enhanced sanitation and hygiene practices, and the introduction of effective vaccines targeting both LT and ST toxins as immediate tools for intervention in endemic settings.

## Data Availability

The original contributions presented in the study are included in the article/Supplementary material, further inquiries can be directed to the corresponding author.
