# Supplementary material for: Epidemiology of enterotoxigenic Escherichia coli among children under five years in Kenya’s urban informal settlement
Source: Front Microbiol. 2025 Aug 15;16:1637369. doi: 10.3389/fmicb.2025.1637369 (PMC12394523; doi:10.3389/fmicb.2025.1637369)
Supplement: Supplementary file 1 [file Data_Sheet_1.pdf]

Supplementary Table 1. Results from Bivariate logistic model (OR)

ETEC in relation to various predictors	OR (95% C.I)	p-value	[95% Conf. Interval
**Age, Months**			
≤12	Ref		
13 - 24	2.564	**0.001**	1.435 - 4.580
25 - 36	2.29	**0.011**	1.211 - 4.327
37 - 48	2.079	**0.033**	1.06 - 4.076
49 - 59	2.429	**0.031**	1.082 - 5.454
**Main composition of households**			
Corrugated Iron	Ref		
Masonry	0.608	**0.025**	0.407 - 0.943
**Main fuel used for cooking by the household**			
Charcoal	Ref		
Gas	0.412	**0.023**	0.192 - 0.883
Kerosene	0.574	0.201	0.245 - 1.344
**Use drum as storage container**			
No	Ref		
Yes	0.604	**0.029**	0.384 - 0.950
**Use Private Flush toilet**			
No	Ref		
Yes	0.313	**0.002**	0.147 - 0 .664
**Household members use water direct from the tap**			
No	Ref		
Yes	0.439	0.083	0.173 - 1.113
**Household members have telephone (mobile phone)**			
No	Ref		
Yes	0.678	0.065	0.448 - 1.025
**Education level of household head**			
College and above	Ref		
Junior high school	1.38	0.267	0.781 - 2.438
Primary school and below	1.763	0.092	0.912 - 3.408
**Kept cattle**			
No	Ref		
Yes	4.924	0.169	0.507 - 47.785
**Has waste container**			
No	Ref		
Yes	0.586	0.119	0.299 - 1.148
**Wash hands before food prep**			
Always	Ref		

Never	0.863	0.783	0.303 - 2.460
Sometime	0.755	0.198	0.492 - 1.159
**Shared pit latrine**			
No	Ref		
Yes	1.462	0.107	0.921 - 2.320
**Use bucket**			
No	Ref		
Yes	0.571	0.115	0.284 - 1.147
**Use jerrican**			
No	Ref		
Yes	1.928	0.175	0.747 - 4.973
**Have refrigerator**			
No	Ref		
Yes	0.391	0.152	0.109 - 1.411
